# Association of Epilepsy Surgery With Changes in Imaging-Defined Brain Age

**DOI:** 10.1212/WNL.0000000000012289

**Published:** 2021-08-10

**Authors:** Christophe E. de Bézenac, Guleed Adan, Bernd Weber, Simon S. Keller

**Affiliations:** From the Department of Pharmacology and Therapeutics (C.E.d.B., G.A., S.S.K.), Institute of Systems, Molecular and Integrative Biology, University of Liverpool; The Walton Centre NHS Foundation Trust (C.E.d.B., G.A., S.S.K.), Liverpool, UK; and Institute of Experimental Epileptology and Cognition Research (B.W.), University of Bonn, Germany.

## Abstract

**Objective:**

To determine whether surgery in patients with mesial temporal lobe epilepsy (mTLE) is associated with reduced brain-predicted age as a neural marker overall brain health, we compared brain-predicted and chronologic age difference (brain age gap estimation [BrainAGE]) in patients before and after surgery with healthy controls.

**Methods:**

We acquired 3D T1-weighted MRI scans for 48 patients with mTLE before and after temporal lobe surgery to estimate brain age using a gaussian processes regression model. We examined BrainAGE before and after surgery controlling for brain volume change, comparing patients to 37 age- and sex-matched controls.

**Results:**

Preoperatively, patients showed an increased BrainAGE of more than 7 years compared to controls. However, surgery was associated with a mean BrainAGE reduction of 5 years irrespective of whether or not surgery resulted in complete seizure freedom. We observed a lateralization effect as patients with left mTLE had BrainAGE values that more closely resembled control group values following surgery.

**Conclusions:**

Our findings suggest that while morphologic brain alterations linked to accelerated aging have been observed in mTLE, surgery may be associated with changes that reverse such alterations in some patients. This work highlights the advantages of resective surgery on overall brain health in patients with refractory focal epilepsy.

Mesial temporal lobe epilepsy (mTLE) is one of the most common forms of focal epilepsy^1^ associated with a number of pathologic alterations linked to premature brain aging.^2^ For one-third of patients with mTLE, antiseizure medication is ineffective,^3^ leading to surgery program referrals that aim to localize and resect the epileptogenic zone.^4,5^ Between 27% and 67% of patients who have surgery become seizure-free.^6^

Despite a risk of cognitive deterioration related to residual function in resected brain tissue, surgery can result in neuropsychologic improvements,^7^ particularly when seizures are controlled and drug load reduced.^8^ The imaging literature provides evidence for postoperative brain network plasticity in support of restorative brain function.^9,10^ However, there is no reliable biomarker to assess the effect of surgery on overall brain health.

A machine learning model for estimating chronologic age from structural MRI scans has shown promise.^11,12^ Increased brain-predicted age (relative to actual age) indicates accelerated aging or higher cumulative exposure/sensitivity to pathologic brain insults, in contrast to brain resiliance.^13^ The brain age gap estimation (BrainAGE) has been used to examine neurodegenerative and psychiatric disorders and the influence of gene interaction, environment, and life burden.^14^ In epilepsy, increased BrainAGE was observed in patients with refractory focal epilepsy^15^ and patients with temporal lobe epilepsy (TLE) and interictal psychosis.^16^

In this study we tested the effect of neurosurgery on BrainAGE as a measure of overall brain health, comparing 48 patients with mTLE before and after surgery to 37 controls. We expected patients to have higher BrainAGE but that successful surgery would be associated with an overall decrease.

## Methods

### Participants

We analyzed structural T1-weighted (T1W) MRI obtained from 48 patients (25 female) with refractory mTLE and neuroradiologically defined unilateral hippocampal sclerosis (HS) (mean age 39.08 years, SD 12.73) who underwent amygdalohippocampectomy at University Hospital Bonn, Germany. Patients (n = 48) were part of a consecutive series of patients (n = 115) being considered for surgery who enrolled in the study and had both pre- and postoperative T1W data suitable for analysis.^18^ After confident diagnosis of unilateral mTLE based on standard clinical protocols and detailed presurgical evaluation, patients underwent selective amygdalohippocampectomy^17^ in either the left (n = 17) or right (n = 31) hemisphere, with subtemporal (n = 21) or transsylvian (n = 27) access.^18^ Presurgical evaluation included interictal EEG with video monitoring and, where clinically required, additional intracranial electrode recording, MRI scanning, and neuropsychologic testing. HS diagnosis was made by a neuroradiologist experienced in epilepsy lesion detection on the basis of hippocampal volume loss and structural alterations observed on the MRI scans.^19^ HS was histologically confirmed in all resected specimens. Follow-up structural T1W scans were acquired for all patients after surgery (mean 1.56 years, SD 0.99, between surgery and the follow-up scan; mean 1.96 years, SD 0.92, between first and second scans). The International League Against Epilepsy (ILAE) outcome classification system was used for postoperative seizure outcome follow-up^20^: 26 were rendered seizure/aura free (SF) (ILAE I) while 22 patients continued to have seizures (persistent seizures [PS]) (ILAE II–VI) following surgery. Clinical data for patient outcome groups is outlined and compared in the table. We also analyzed MRI from a sample of 37 neurotypical controls similar in age and sex to the individuals who underwent surgery for epilepsy (mean age 40.08 years, SD 13.94; 21 female).

**Table:**
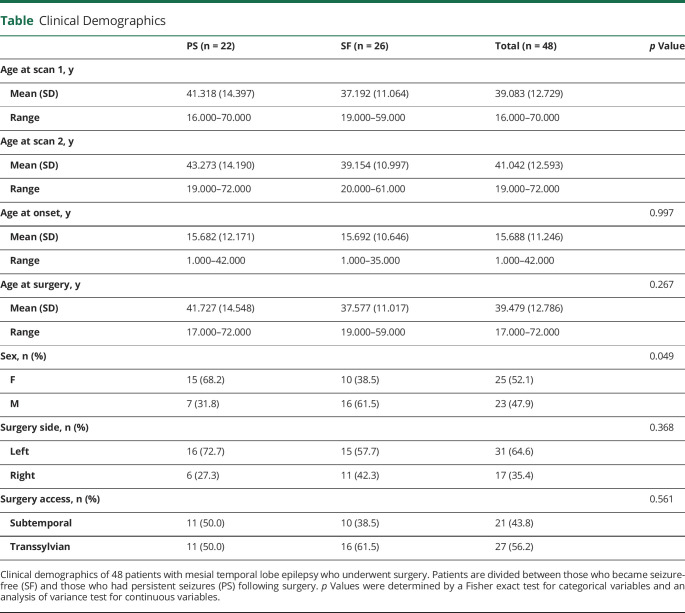
Clinical Demographics

### Imaging

All participants were scanned on a 3T scanner (Magnetom Trio, Siemens) and an 8-channel head coil at the Life & Brain Center in Bonn. Three-dimensional T1W magnetization-prepared rapid gradient echo images were used for the BrainAGE analysis (160 slices, repetition time 51,300 ms, inversion time 5,650 ms, echo time 53.97 ms, resolution 1.0 × 1.0 × 1.0 mm^3^, flip angle 10°).

### Brain Age Prediction

Brain-predicted age was computed from raw T1W MRI scans using the BrainAgeR analysis pipeline (github.com/james-cole/brainageR), previously described in detail.^12^ The pipeline includes the segmentation and normalization of MRI with SPM12's DARTEL toolbox.^21^ The quality of tissue segmentation was systematically assessed for all participants and no errors were found through visual inspection of segmentation output. After CSF segmentation masking, preprocessed gray and white matter images were vectorized and concatenated. These data were then entered into a principal components analysis to reduce dimensionality. Components for the top 80% of variance were used (n = 435) for brain age prediction in a machine-learning algorithm based on a pretrained Gaussian process regression model implemented in R package Kernlab.^22^ This model was trained on scans of 3,377 healthy individuals from 7 publicly available datasets^12^ and tested on 611 different scans of healthy individuals aged between 18 and 90 years. The model accurately predicted chronologic age (*r* = 0.95, *R*^*2*^ = 67.24%, MAE 4.9 years). As with other brain age models, a proportional bias was observed where chronologic age correlated with the difference between brain predicted and actual age (*r* = −0.379).^23^

### Brain Change Control

To control for between-patient differences in overall brain volume change following surgery we used SIENA, part of FSL.^24^ Brain and skull images were first extracted from the 2-timepoint whole-head input data. The 2 brain images were then aligned to each other and resampled into a space halfway between the two. Tissue segmentation was then performed to identify nonbrain/brain edge points.^25^ The perpendicular displacement of edges between the 2 timepoints was then estimated at these edge points. Using mean edge displacement, a whole brain estimate of percentage brain volume change (PBVC) between the 2 timepoints was computed.

To ensure that the surgical cavity did not significantly bias the BrainAGE measurement, we also implemented an automated lesion-filling procedure^26^ previously tested in relation to BrainAGE in a large (n > 500) multiple sclerosis cohort.^27^

### Statistical Analysis

Statistical analyses were performed in R (v 3.6.0). Fisher exact test was used for categorical variables and an analysis of variance test for continuous variables to compare demographic differences between clinical outcome groups (table). All were nonsignificant (*p* > 0.05), with the exception of sex, which was included as a control variable along with age in subsequent statistical modeling.

**Figure 1:**
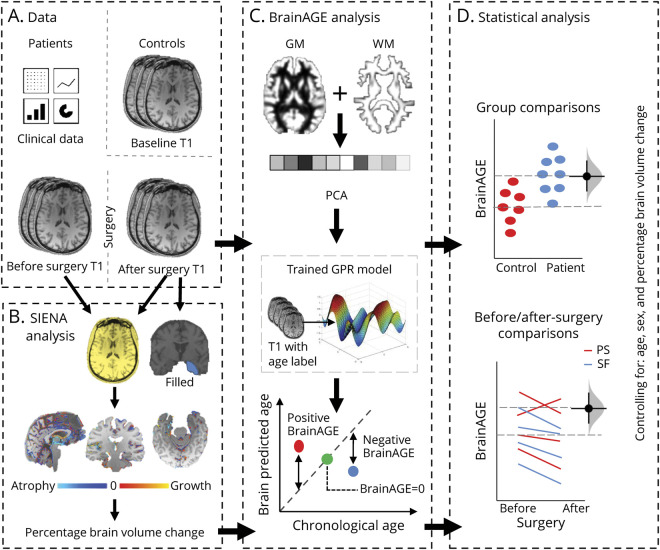
Graphic Description of the Analysis Pipeline (A) In addition to clinical patient data, 3D T1-weighted MRI scans were acquired for 48 patients with mesial temporal lobe epilepsy before and after temporal lobe surgery and for 37 controls. (C) Brain age was estimated for each scan using a trained gaussian processes regression (GPR) model following tissue segmentation, vectorization, and principal components analysis (PCA)–based dimension reduction. Brain age gap estimation (BrainAGE) was computed as brain-predicted age minus actual age. BrainAGE comparisons were made between patients and control groups before and after epilepsy surgery, controlling for percentage brain volume change (SIENA; B), age, and sex (D). GM = gray matter; WM = white matter.

BrainAGE was calculated as the brain-predicted age minus chronologic age at the time of the MRI scan. As there was no evidence of non-normal distribution (W = 0.98, *p* = 0.150) and inhomogeneity of variance between groups (Flinger-Killeen: med χ^2^[2] − 1.43; *p* = 0.489) in the data, we used a linear multiple regression model to compare BrainAGE between controls and patient groups (SF, PS, left mTLE, right mTLE) both before and after surgery. Age was included as a covariate in all models to correct for the previously reported proportional bias,^28^ in addition to sex, gray matter, white matter, and CSF brain volume. Figure 1 is a graphic representation of the study analysis pipeline. To directly compare BrainAGE before and after surgery within patient participants and across patient groups, we used a repeated-measures mixed model with patients’ ID included as a random effect and PBVC, resection size, age, sex, and brain tissue volumes as control variables.

### Standard Protocol Approvals, Registrations, and Patient Consents

All patient and control participants provided written informed consent and the local ethics committee approved this study.

### Data Availability

The data that support the findings of this study are available via the corresponding author, on reasonable request.

## Results

**Figure 2:**
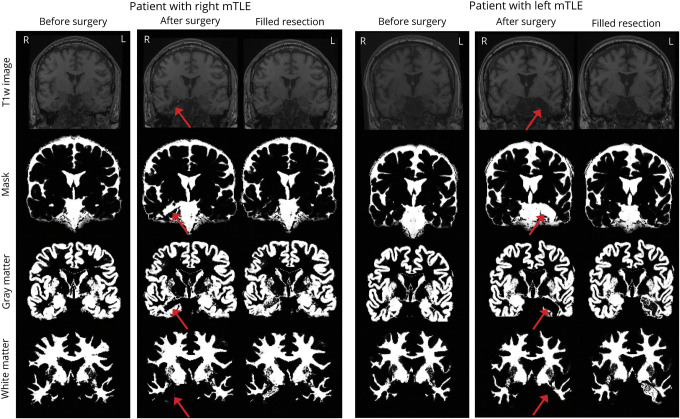
T1-Weighted Images Before and After Surgery for 2 Patients, 1 With Right- and the Other With Left-Lateralized Mesial Temporal Lobe Epilepsy (mTLE) Below the associated masks, CSF and gray and white matter segmentations used in the computation of brain age gap estimation (BrainAGE) are included for original (unfilled) and filled images following surgery. Resection location is indicated by a red arrow for original images used in the analysis.

In accordance with the findings of previous work,^27^ we found that the difference between BrainAGE for unfilled and filled postoperative scans was also not significant in our data (mean difference −0.79, 95% confidence interval [CI] −5.66 to 4.09, *t*[37] −0.33, *p* = 0.745), suggesting that the pipeline is robust to surgical cavity–related bias. Figure 2 shows T1W images (first row) before and after surgery for 2 patients, one with right and the other with left-lateralized medial mTLE. The associated mask (CSF) and gray and white matter segmentations used in the computation of BrainAGE are also included for original (unfilled) and filled images. As the figure indicates, resections were appropriately masked out (see row 2) of gray and white matter segmentations in original images for all patients. Resections in filled images tended to be included in white matter segmentations. We therefore used BrainAGE computed from original images for the analysis.

Brain predicted age was highly correlated with chronologic age (*r* = 0.91, 95% CI 0.83–0.95) in controls with a mean absolute error of 4.08 years, comparable to the MAE found in the original training datasets (MAE 4.9).^12^ The mean (±SD) BrainAGE in controls was −0.68 (±5.85) years. There was a significant correlation between BrainAGE and chronologic age in controls (*r* = −0.45, 95% CI −0.67 to −0.14), in line with the previously reported proportional bias.^28^

**Figure 3:**
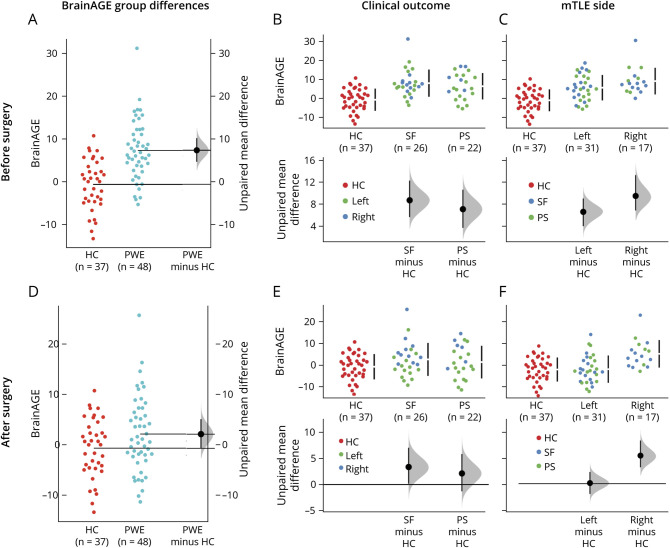
Between-Group Comparisons of Brain Age Gap Estimation (BrainAGE) Comparisons of BrainAGE (computed as brain-predicted age minus actual age) for patients with epilepsy (PWE) and healthy controls (HC) (A, D), patients who are seizure-free (SF) and those with persistent seizures (PS) following surgery (B, E), and left- and right-sided mesial temporal lobe epilepsy (mTLE) (C, F). Comparisons are presented with BrainAGE values before (A–C) and after (D–F) surgery. In A and D, raw data points for BrainAGE in HC and PWE are shown in the left panel with unpaired group difference estimations plotted as a bootstrap sampling distribution (n = 5,000) (shaded area). Average effect size (mean difference) is depicted as a black dot and the 95% confidence interval (CI) indicated by the ends of the vertical error bar. In B, C, E, and F, raw data for patient subgroups are compared to HC (red) and plotted/color coded on the upper panel with associated estimation plots shown below. Note that where the 95% CIs (error bars) cross the horizontal line at zero, an effect size equal to zero is possible, i.e., no reliable difference from HC.

Figure 3 shows group differences with estimation graphics implemented in dabestr.^29^ Data points are displayed as a swarmplot with effect size presented on an aligned axes as a 95% CI calculated through bootstrap sampling (n = 5,000). On average, patients with epilepsy showed an increased BrainAGE of 7.97 years compared to controls prior to surgery (95% CI 5.26–10.8) and an increase of 2.8 years following surgery (95% CI 0.05–5.78). BrainAGE differences between controls and patient subgroups before surgery were as follows: SF 8.71 (95% CI 5.65–12.3); PS 7.09 (95% CI 3.69–10.6); left mTLE 6.73 (95% CI 3.58–9.66); right mTLE 10.2 (95% CI 7.03–14.8). BrainAGE differences between controls and patient subgroups after surgery were as follows: SF 3.37 (95% CI 0.161–7.07); PS 2.13 (95% CI −1.31 to 5.86); left mTLE 0.16 (95% CI −2.78 to 3.17); right mTLE 7.61 (95% CI 4.45–11.7). Plots in figure 3 show increased BrainAGE in patient groups (PWE, SF, PS, left, right) compared to controls before but not after surgery, with greatest postoperative reduction occurring for patients with left mTLE.

The linear regression model used to evaluate group differences in baseline BrainAGE (before surgery and correcting for age and sex, gray matter, white matter, and CSF brain volume) explained a significant and substantial proportion of variance (*R*^2^ = 0.60, 90% CI 0.44–0.68, 
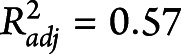
). Significantly higher BrainAGE was found for both SF (b = 5.47, 95% CI 2.70–8.25) and PS (b = 4.69, 95% CI 1.87–7.50] patient groups compared to controls. However, the estimation marginal means^30^ indicated that BrainAGE difference between SF and PS was not significant (*t*[76] = 0.52, *p* = 0.862). Patient outcome groups were also not significantly different from controls using postoperative BrainAGE values (model fit = *R*^*2*^ = 0.50, 90% CI 0.32–0.59, 
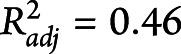
; SF = b = −0.07, 95% CI −2.90 to 2.77; PS = b = −0.17, 95% CI −3.05 to 2.71). Although resection size did not differ between patients with left- and right-lateralized mTLE (t[44] = −0.03, *p* = 0.976), grouping patients by lateralization again revealed higher BrainAGE before surgery for both groups compared to controls (left mTLE = b = 3.72, 95% CI 1.23–6.21; right mTLE = b = 7.57, 95% CI 4.62–10.52) and a significant left vs right difference (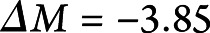, 95% CI −7.39 to −0.31). Following surgery, however, patients with right (b = 7.46, 95% CI 3.83–11.09) but not left (b = 0.25, 95% CI −2.72 to 3.23) mTLE had significantly higher BrainAGE compared to controls, with marginal means estimation showing right vs left difference to be more significant in postoperative BrainAGE (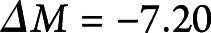, 95% CI −11.71 to −2.70).

**Figure 4:**
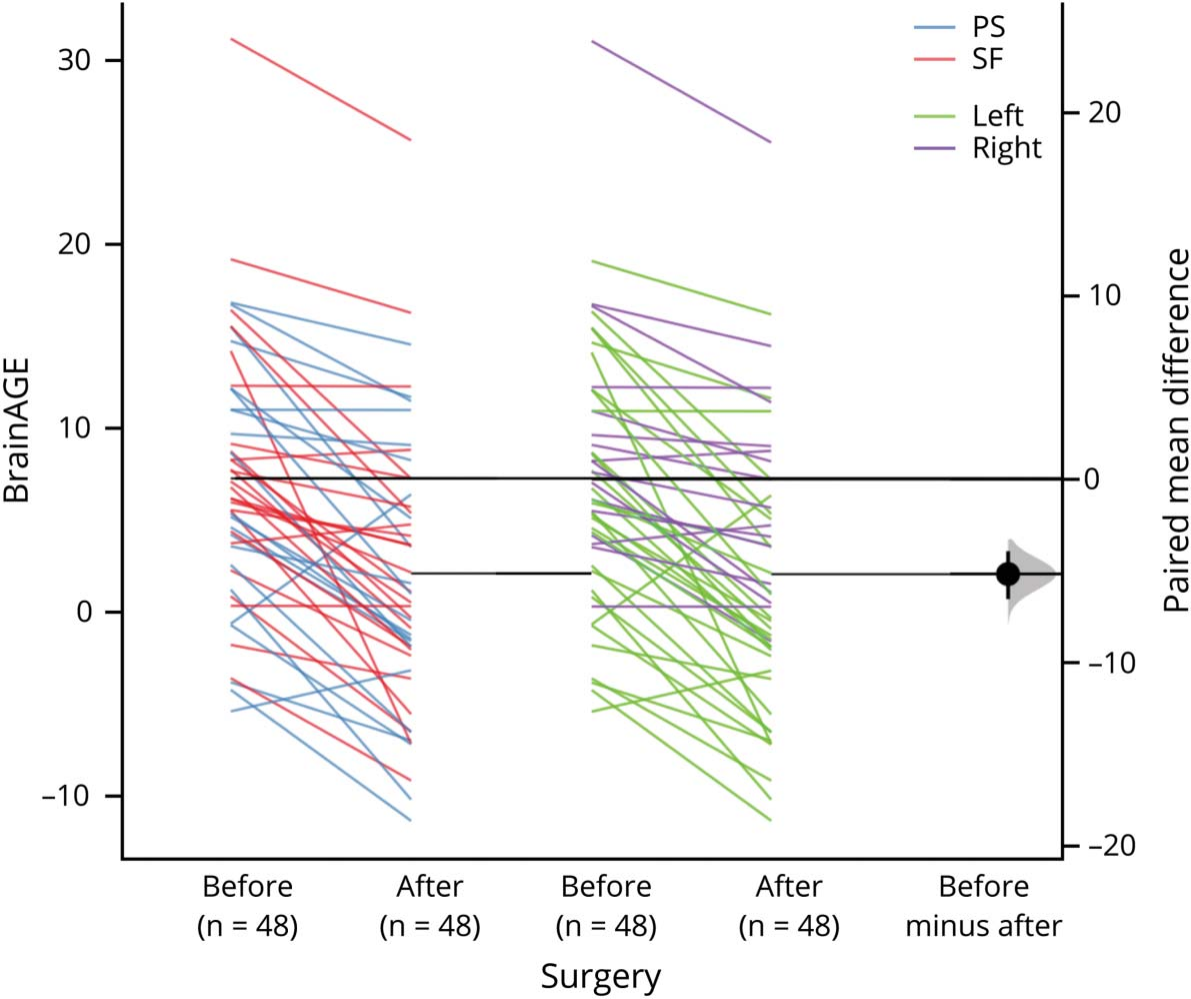
Effects of Epilepsy Surgery on Brain Age Gap Estimation (BrainAGE) Paired difference of BrainAGE (computed as brain-predicted age minus actual age) before and after epilepsy surgery in patients with mesial temporal lobe epilepsy (mTLE). Colors indicate patient subgroups (PS = persistent seizures; SF = seizure-free; left = left mTLE; right = right mTLE). Paired differences are shown on the right with confidence plotted as a randomized bootstrap sampling distribution (n = 5,000) (shaded area) and the average effect size depicted as a black dot and the 95% confidence interval indicated by the ends of the vertical error bar.

Figure 4 shows paired mean difference estimation plots of BrainAGE for different patient groups. The average difference after compared to before surgery was −5.17 years (95% CI −6.53 to −3.91). The total explanatory power of the repeated-measures mixed model used to assess the effect of surgery on BrainAGE was substantial (conditional *R*^*2*^ = 0.87) and the part related to the fixed effects alone (marginal *R*^*2*^) was 0.31. As can be seen in the paired difference plot presented in figure 4, the main effect of surgery was large with BrainAGE significantly higher (M = 5.17 years) before compared to after surgery (β 7.34, SE 0.97, standardized β 0.94, *p* < 0.001). The effect of outcome (SF–PS) was not significant (β −0.70, SE 1.67, standardized β −0.09, *p* = 0.677), although a trend emerged when only patients with continuing seizures with loss of awareness (ILAE 3–6) were included into the PS group (β −3.05, SE 1.61, *p* = 0.065). The interaction effect of surgery and outcome was not significant (PS = ILAE 2–6: β −0.80, SE 1.24, standardized β −0.10, *p* = 0.519; PS = ILAE 3–6: β −0.39, SE 1.35, standardized β −0.05, *p* = 0.773). The main effect of lateralization (right/left mTLE/surgery) was large and significant with an overall increased BrainAGE associated with right lateralization (β 8.05, SE 1.58, standardized β 1.05, *p* < 0.001). The interaction between surgery (BrainAGE before and after) and lateralization was also significant, indicating significantly less postoperative BrainAGE reduction for right compared to left mTLE (β −3.71, SE 1.22, standardized β −0.48, *p* < 0.01). The main effect of resection size on BrainAGE was not significant (β 0.75, SE 0.80, standardized β 0.10, *p* = 0.347) and there was a small positive effect of PBVC (β 1.00, SE 0.48, standardized β 0.21, *p* < 0.05). Other variables such as age at seizure onset, years from onset to surgery, surgery access, and seizure frequency and burden were removed from the final model due to minimal and nonsignificant effects on BrainAGE.

## Discussion

We used a brain-predicted age measure to investigate the effects of epilepsy surgery on overall brain health. Preoperatively, patients showed an increased BrainAGE (difference between brain predicted and actual age) of more than 7 years compared to controls. However, surgery was associated with a BrainAGE reduction of an average of 5 years irrespective of whether the procedure resulted in seizure freedom. This postoperative reduction was particularly pronounced for patients with left lateralized mTLE, where BrainAGE values following surgery resembled those of controls.

Given the correlation between BrainAGE and cognitive decline,^31^ the postoperative normalization of BrainAGE is consistent with literature that shows restoration of some aspects of neuropsychologic function following successful epilepsy surgery. This has been observed in domains including verbal fluency, IQ, executive functioning, and attention, despite an increased risk to verbal memory associated with residual function in resected regions.^7,8^ The imaging literature also provides evidence for neuroplastic network changes with restorative potential.^9,10^ In a longitudinal fMRI study, plasticity of a working memory network was observed after temporal lobe surgery.^9^ Another study examining functional connectomes in the brainstem found that connectivity patterns of patients were more likely to resemble those of controls after epilepsy surgery.^10^ Other research, however, shows a limited effect of the procedure on functional and structural brain networks,^32,33^ and that connectivity normalization is associated with whether or not a patient becomes seizure-free as a result of neurosurgery.^34,35^

We expected that patients who were rendered completely seizure-free (ILAE 1) would have lower BrainAGE compared to patients with persistent postoperative symptoms (ILAE 2+). However, there was no significant difference between clinical outcome groups. Postoperative cognitive and quality-of-life outcomes are influenced by many factors and only partly depend on seizure control.^36^ BrainAGE may therefore reflect surgery-related changes that are not directly associated with seizure status. Furthermore, following the majority of previous epilepsy cohort studies,^18^ we used a dichotomous outcome grouping (ILAE 1 vs ILAE 2+) that does not account for the considerable cumulative reduction in seizures for patients in the ILAE 2+ (PS) group. Notably, we did find a borderline difference between patients rendered seizure-free and only those who continued to experience debilitating postoperative seizures (ILAE 3–5), suggesting that there may in fact be a subtle relationship between BrainAGE and seizure outcome that may be difficult to detect in relation to surgery due to the personal improvements in seizure control that patients are likely to experience following surgery (an ILAE 3 patient who previously had a seizure a day will be a relative clinical success). Although we did not have access to patients' seizure outcome in response to antiseizure medication before surgery, it may be that BrainAGE reduction is more closely related to the difference between seizure outcome after compared to before surgery. Without information on postoperative antiseizure medication, it was also not possible to disentangle the effects of surgery from medication on seizure outcome. However, this does not have implications for our findings, as medication is not likely to have been significantly altered during the short period between pre- and postoperative scans (mean 1.96 years, SD 0.92).

Although we controlled for postoperative volume change and resection size in our statistical modeling, we cannot exclude the possibility that BrainAGE reduction may be a by-product of invasive surgery and unrelated to increased brain resilience. However, a supplementary analysis revealed that there was no significant BrainAGE difference between postoperative scans with artificially filled and unfilled resections, in accordance with a previous large-scale multiple sclerosis study,^27^ suggesting that the measure may capture features associated with brainwide correlates of surgery beyond those relating to presence of the surgical cavity. The link between postsurgery BrainAGE reduction and brain resilience could be more reliably established in future studies if a strong relationship between BrainAGE reduction and positive cognitive and quality of life outcomes is observed in a study with more postoperative scans to maximize the generalizability of study results. It may also be possible to isolate the BrainAGE correlates of surgery specifically related to TLE through comparison with a patient group without TLE that have undergone equivalent surgery treatment.

More generally, our results support the proposal that mTLE is related to morphologic changes of accelerated aging, in accordance with evidence associating the disorder with neurodegenerative features such as neuronal loss in and around the disease epicenter,^37,38^ axonal sprouting,^39^ blood–brain barrier leakage,^40^ loss of brain plasticity and reserve capacity,^41^ and increased inflammation.^42^ The extent of the BrainAGE increase (7.9 years) in patients before surgery found in our study is also consistent with previous epilepsy brain age studies that show comparable effects of between 4.5 and 10.9 years in focal epilepsy cohorts.^15,16^ These findings are in line with clinical and epidemiologic studies that highlight the benefits of earlier epilepsy surgery interventions over prolonged medical therapy.^43-45^

Our findings showed that patients with left mTLE were more likely to have normalized BrainAGE after surgery relative to patients with right mTLE, although no difference was found in resection size between left and right surgery. This is an additional novel finding and we can only speculate on the reasons why there is a lateralization difference. Surgery aside, it is well demonstrated that patients with left and right mTLE have different distributions of brain abnormalities: patients with left mTLE are frequently reported to have a more bilateral and widespread distribution of brain alterations relative to patients with right mTLE^46^ as well as a more intense progression of white and gray matter atrophy.^47^ A systematic review of neuropsychologic outcomes after epilepsy surgery showed that the greatest rate of improvement across all domains occurred in verbal fluency with left-sided temporal surgery.^7^ It has also been reported that surgery reduces mortality associated with refractory mTLE but only in patients with left-sided surgery.^48^ The same work indicated that mortality was not related to postoperative seizure outcome. In fact, there is little evidence to suggest that side of surgery is related to seizure outcome. Given the established link between BrainAGE and increased mortality,^12^ our findings, which should be taken as preliminary, leave open the possibility that surgery may have particular benefits for patients with left mTLE, although confirmation would require a targeted investigation that also measures surgery-related neuropsychologic changes.

BrainAGE algorithms are likely to improve in the future using larger training datasets and by taking advantage of multimodal imaging (e.g., T2- and diffusion-weighted MRI sequences). It is also not yet clear what 3D T1W image features most reliably contribute to the BrainAGE measure and further methodologic work in neurotypical and clinical populations is needed to better understand how it reflects the nonlinear patterns of age-related changes including regional brain volume reductions. Given that there is a known pattern of cerebral (particularly limbic) atrophy in TLE,^1,2^ and that decreasing brain volume is a characteristic of aging,^31^ then increasing atrophy in TLE may drive greater BrainAGE. However, the factors that suggest a recovery of increasing BrainAGE after surgery is unknown.

Reliance on largely standardized MRI allows the method to be widely applicable in clinical settings where the sequence is acquired for all patients referred to epilepsy surgery programs. Based on our findings, brain-predicted age models have the potential to further risk stratify patients who will benefit from epilepsy surgery, thereby improving the personalized medicine approach for people with refractory epilepsy in conjunction with other imaging and neuropsychologic screening tools.^14^ Furthermore, given that increased BrainAGE is an independent predictor of mortality,^12^ the imaging biomarker may be used to identify patients at high risk of sudden unexplained death in epilepsy. Brain age models may play both a prognostic and diagnostic role in the neurocognitive and psychiatric disorders associated with epilepsy as these have proven to be useful in the context of psychiatric disease and impaired cognition.^11,49^ Other advances in deep learning applied to brain images are beginning to play a role in clinical decision-making, such as in the automatic classification and prognostics of TLE.^50^

Our study found that epilepsy surgery was associated with reduced brain imaging–defined age, suggesting that some morphologic brain changes linked with accelerated aging in mTLE may be reversible. This is consistent with studies that found neuropsychologic improvements following surgery and those calling for earlier surgical intervention where medical therapy is ineffective. Models of brain-predicted age may provide insight into the treatment and prognosis of epilepsy.

## Glossary

BrainAGE: brain age gap estimation
CI: confidence interval
HS: hippocampal sclerosis
ILAE: International League Against Epilepsy
MAE: mean absolute error
mTLE: mesial temporal lobe epilepsy
PBVC: percentage brain volume change
PS: persistent seizures
SF: seizure/aura-free
T1W: T1-weighted
